# PACEMweb: a tool for aggregate consumer exposure assessment

**DOI:** 10.1038/s41370-022-00509-7

**Published:** 2022-12-15

**Authors:** Christiaan J. E. Delmaar, Roel Schreurs, Martine I. Bakker, Jordi Minnema, Bas G. H. Bokkers

**Affiliations:** National Institute for Public Health and the Environment—RIVM, Bilthoven, The Netherlands

**Keywords:** Aggregate Exposure, Consumer products, PACEM, Exposure modelling, Chemicals

## Abstract

**Background:**

To ascertain the safe use of chemicals that are used in multiple consumer products, the aggregate human exposure, arising from combined use of multiple consumer products needs to be assessed.

**Objective:**

In this work the Probabilistic Aggregate Consumer Exposure Model (PACEM) is presented and discussed. PACEM is implemented in the publicly available web tool, PACEMweb, for aggregate consumer exposure assessment.

**Methods:**

PACEM uses a person-oriented simulation method that is based on realistic product usage information obtained in surveys from several European countries. PACEM evaluates aggregate exposure in a population considering individual use and co-use patterns as well as variation in product composition. Product usage data is included on personal care products (PCPs) and household cleaning products (HCPs).

**Results:**

PACEM has been implemented in a web tool that supports broad use in research as well as regulatory risk assessment. PACEM has been evaluated in a number of applications, testing and illustrating the advantage of the person-oriented modeling method. Also, PACEM assessments have been evaluated by comparing its results with biomonitoring information.

**Significance:**

PACEM enables the assessment of realistic aggregate exposure to chemicals in consumer products. It provides detailed insight into the distribution of exposure in a population as well as products that contribute the most to exposure. This allows for better informed decision making in the risk management of chemicals.

**Impact:**

Realistic assessment of the total, aggregate exposure of consumers to chemicals in consumer products is necessary to guarantee the safe use of chemicals in these products. PACEMweb provides, for the first time, a publicly available tool to assist in realistic aggregate exposure assessment of consumers to chemicals in consumer products.

## Introduction

People are continuously exposed to chemicals in the consumer products they use. Such consumer products include personal care products (PCPs), household cleaning products (HCPs), textiles, plastics, etc. To assess the risk associated with exposure to chemicals in consumer products, an evaluation of exposure is needed. A specific chemical may be contained in multiple products, requiring the assessment of total, or aggregate exposure from these products combined.

Although occasionally the need for aggregate exposure assessment is acknowledged in regulatory risk assessment, generally very crude methods of aggregate exposure assessment are employed. For example, in the EU, the Scientific Committee for Consumer Safety (SCCS) [1] recommends calculating the aggregate exposure by adding exposures from 17 individual cosmetic product types, assuming maximum concentration levels in each product. Such methods typically lead to unrealistically high estimates of exposure. They may help to demonstrate that safe use of the chemical in products is plausible, if the estimated exposure is below some health-based limit value, even under these extreme assumptions. However, if the health-based limit value is exceeded, the exposure and risk assessments are inconclusive: it remains unknown whether the exposure is truly too high or only exceeds the limit value due to the underlying conservative assumptions.

More realistic exposure assessment methods need to consider patterns of use and co-use of consumer products in a population, acknowledging that not all individuals use all products and that not all brands of a product will contain the chemical, or at least not to the maximum concentration level. Information on the quantities and frequencies of use of various product groups has become available in recent decades [2–11]. To account for these aspects, person-oriented modeling is required [12–14]. Person-oriented modeling approaches have been described [15] and implemented in e.g., the Creme model [10]. Creme however, is a commercial product that is not publicly accessible to the risk assessment community, neither for use nor for review. Also publicly available is the SHEDS-HT tool [16], but this tool is developed specifically to simulate consumer exposures for United States populations and its applicability to e.g., the European context is unknown.

For use in the public domain and, in particular for regulatory and research applications, the Probabilistic Aggregate Consumer Exposure Model (PACEM) has been developed. PACEM incorporates information on product usage for personal care products (PCPs) and household cleaning products (HCPs) that was obtained in different surveys [2, 3, 11, 17]. It combines the simulation of realistic product use patterns with information on substance concentrations in the products. In particular, as information on the co-use of different products by each individual is available from the surveys, realistic simulation of the combined use of multiple products by each individual can be conducted.

PACEM has been applied in a number of exposure assessments. Gosens et al. [18] used a prototype version of PACEM to estimate exposure of young children (age 0–3) to various parabens in PCPs. They demonstrated the benefits of using a person-oriented modeling method as used in PACEM over the use of a deterministic addition method, providing a much more informed exposure assessment that allowed for a more balanced interpretation of the risks involved in the exposure.

In Delmaar et al. [19] and Dudzina et al. [20] exposure assessments for PCPs containing diethyl phthalate (DEP) and decamethylcyclopentasiloxane (D5) respectively, were compared to biomonitoring data, demonstrating that PACEM assessments and biomonitoring data were consistent.

Ezendam et al. [21] Nijkamp et al. [22] Jongeneel et al. [23], and Garcia-Hidalgo et al. [24] applied PACEM in risk assessments of dermal sensitizing substances in PCPs and HCPs. In particular, Jongeneel et al. [23] demonstrated how a refined person-oriented approach may be used to evaluate how different sources (in this case PCPs and HCPs) contribute to a health problem, providing very direct information on risk mitigation policies.

Until recently, the PACEM model was only available as a database and model-script in the R simulation language [25]. In that form it was only usable by exposure modeling experts. In 2022, an implementation of the model in a user-friendly web tool has become available. PACEM includes product usage data on PCPs and HCPs from three different surveys. To assess exposure to a specific substance, user input on product concentrations of the substance as well as event exposure information is required. The web tool provides a user interface to the model and is freely accessible at www.pacemweb.nl.

Here, we describe in some detail the PACEM modeling method, implementation aspects and use of the web tool. Finally, we describe an illustrative case study conducted with the web tool, the exposure assessment of methylisothiazolinone (MI), based on newly acquired concentration data of MI concentrations in PCPs and HCPs. MI has been identified as a common cause of allergic contact dermatitis, while being commonly used in consumer products as a preservative. This case study exemplifies the need of using a realistic aggregate exposure model as provided by PACEM, as opposed to using conservative, worst-case exposure models.

## Model structure

### Product usage data

PACEM is based on product usage information in different populations to derive the exposure to substances in these products: two surveys describing PCP usage, and one on HCPs. The PCP surveys are designated as the ‘Dutch PCP’ survey and the “European PCP” survey. The HCP survey is referred to as the “European HCP” survey. The surveys were conducted using different methods. Data obtained in the surveys needed to be converted to a format usable in PACEM simulations.

The Dutch PCP survey [11] reported the frequency of use and the amount of 32 PCPs among 516 adult (age 18–70) men and women from the Netherlands. General information of the participants was recorded, i.e., their body weight, age, sex, level of education, skin type, skin color, smoking habits and alcohol use. The participants indicated their frequency of use of each of the 32 PCPs by selecting one of the provided frequencies: <1, 1–2, 3–4, 5–6 times per week or 1 or ≥2–3 times per day. The amount of product used per application was surveyed using photographs that visualized different amounts of product used. The photographs contained three images displaying an increasing amount of product. In addition, information was recorded on the type and brand of the product, the application area on the body, the time of day a product was used (e.g., morning or evening), the location of use (indoors or outdoors) and the presence of ventilation. For the application in PACEM, the use frequency information was quantified by assigning a uniform probability to the use frequency within each frequency interval specified in the categorical data, and random sampling from the resulting probability distribution. Similarly, the categorical use amount data from the survey was converted to a uniform within-category distribution with ranges corresponding to amounts as measured from the photographed situations. When performing simulations in PACEM, a specific use frequency and use amount are sampled from these distributions. Thus, for example, if a survey respondent reported use frequency of “1–2 times per week” and use amount of “3–4 g”, a specific use frequency in events/week is sampled from a Uniform(1,2) distribution, and a specific use amount in g/event is sampled from a Uniform(3,4) distribution.

The European PCP survey was constructed from multiple underlying studies surveying the use frequency and use amount. The Toiletries and Cosmetics Database (ETCD-1) [2, 3] provided information on the use frequency of PCPs for France, Germany, UK and Spain. In each country the study inclusion criteria were as follows: Both females and males, age 17–74 years, demographically representative sample, including working status and age, habitual users of all products, brands and categories for toiletries and cosmetics. In total, data from 23,232 volunteers were obtained. Product use was reported using a diary for 7 consecutive days in 1 or 2 nonconsecutive weeks. Personal information recorded included country, age, size of household, sex, working status, family composition, hair and skin type, and BMI. For each product, the week day of use, time of use and body part of application were recorded. The survey included 21 products (Table S1). Information on the used amount of product was obtained in two separate surveys of 997 volunteers in total, age 17–74. Participants completed the full 2 week usage period of their own products, which were weighed at the start and end of the study. Using information on the number of uses in the study period, the average amount of product per use per event was obtained.

The European HCP data was obtained from the EPHECT study [17, 26]. The EPHECT study collected product usage data in ten different European countries in the population aged 18 and older. The survey recorded categorical frequency of use. e.g., daily, once a week, several times per week, etc., which were transformed to daily frequencies (Supplementary information). Quantity of product used (quantified in different measures such as: quantity of liquid, number of sprayings, number of wipes etc.) and dilution patterns. This information was converted to amounts in grams (Supplementary information). Data from Germany, France, UK and Spain, were included in PACEM as these corresponded to countries surveyed in the European PCP survey. Products surveyed included all-purpose cleaners, kitchen cleaners, floor cleaners, glass and window cleaners and bathroom cleaners. For all products, different product forms were distinguished (cream, foam, gel, liquid, powder, spray, tablets and wipes).

Information on the surveys is summarized in Table S2.

### Modelling aggregate exposure in PACEM

**Fig. 1:**
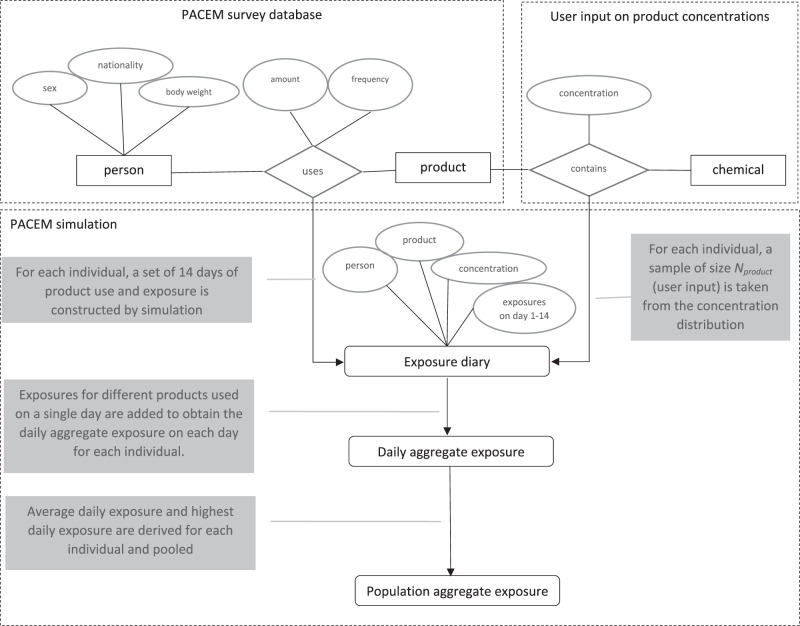
Flow of the PACEM simulation. The PACEM survey database contains information on persons surveyed and their product usage. In an exposure simulation for a specific chemical, the survey is combined with user input information on the concentration of the chemical in consumer products. Product usage data and concentration information is combined by random sampling of the concentration information and assigning sets of sampled products with a specific concentration to each individual in the survey population. Next, product use and exposure for a period of 14 days is simulated for each individual. Finally, from the simulated product exposures on each day, aggregate exposure is determined by adding up exposures on the day, resulting in a set of 14 daily aggregate exposure estimates for each individual in the model population.

Aggregate exposure in PACEM is calculated by simulating a 14 day period of product use for each individual in a population, and determining the daily total exposure from all products used by each individual on each particular day. The procedure results in a sample of individuals and their daily exposures in the simulated period. In detail, the following steps are taken (see also Fig. 1):

1. All individuals in the selected survey are duplicated into a model population, retaining the personal information (e.g., body weight, sex) as well as the product use profile (which products, frequency and amount of use).
2. For each individual a set of 14 days of product usage is constructed. For the European PCP data, the entire set of days is copied from the database on the product usage data. For European HCP and Dutch PCP data, the days are constructed by random sampling from a Bernoulli distribution Bernoulli(p), in which p, the probability of use on a particular day, is derived from the use frequency information. For the amount used probability distributions in the product usage database are sampled for each use in the set of simulated days separately.
3. The product use profiles of all individuals are then combined with product concentration information of the substance. Products with a particular sampled concentration are assigned to each (person, product) combination according to:
  1 $$concentration = {\it{Bernoulli(occurrence)}} \times {\it{P}}\left( {{{{{{{{\mathrm{concentration}}}}}}}}} \right)$$
  Where “occurrence” is the fraction of the products available on the market that actually contain the substance of interest, and P(concentration) is the probability distribution representing the (variation in) concentration in products that contain the substance. PACEM supports different parametric probability distributions for the concentration, including point, uniform, normal, lognormal and triangular distributions. Product concentration information is user input in PACEM.
4. For each individual, a sample of size N_product_ (also user input) is taken from the concentration distribution. This sample represents the fact that individuals with a similar use profile use products with different substance content (e.g., different brands). In other words, each modeled individual represents a group of different persons in the population that vary in the brand (and hence substance concentration) of product they use, but have otherwise similar usage patterns (in terms of frequency and amount). For each concentration in the sample, exposure is calculated (details below), resulting in a set of N_product_ exposure values for a single individual, for each product, on each day.
5. Exposures for different products used on a single day are added to obtain the daily aggregate exposure on each day for each individual. Here, individual samples for different products are randomly paired. This results in a set of N_product_ samples of aggregate exposure for each person on each of the simulated 14 days.
6. From set of N_product_ daily exposures of all individuals in the modeled population, two different summary exposure characterizations are derived: the average daily exposure over the 14 days and the highest daily exposure in the set of 14 days of each sample in the set. This results in N_product_ estimates per individual of both the average and highest daily exposure. The average exposure reflects long-term exposure, whereas the highest day of exposure reflects the short-term or acute exposure.
7. Finally, the distribution of aggregate exposure in the population is represented graphically as a histogram or cumulative distribution plot, or summarized as sets of percentiles.

To characterize exposure, two different metrics are supported in PACEM:

1. “dermal load”, defined as the amount of chemical substance on the skin per cm^2^ exposed skin. The dermal load is generally deemed most appropriate for the risk assessment of skin sensitization by a substance [26, 27].
2. “systemic exposure”, defined as the amount of chemical absorbed per kg body weight.

Dermal load is calculated as

2 $${\it{Dermal}}\,{\it{load}} = \frac{{[amount] \times [{\it{concentration}}] \times [{\it{retention}}]}}{{[skin\,surface\,area]}}$$

Here, the amount is the amount of product used, concentration the concentration of the substance in the product, retention is a factor between 0 and 1 that accounts for the fact that only part of the used product may end up and remain on the skin (e.g., the fraction remaining after washing the skin). PACEM distinguishes different body parts: trunk, head, arms, hands, legs, and feet. For each of these body parts, different retention factors may be provided by the user. Aggregate exposure is determined per body part. The body part with the highest dermal load is taken as the measure of exposure.

Systemic exposure is calculated as

3 $$Exposure = \frac{{[amount] \times [concentration] \times \mathop {\sum }\nolimits_{route} (EF \times fabs)_{route}}}{{[body\,weight]}}$$

Here, EF_route_ represents the so-called “exposure fraction” of a specific route. It is defined as the fraction of the substance used with the product to which a consumer is exposed. It accounts for the fact that a person will generally not be exposed to all the substance that he uses. The exposure fraction is user input. It is discussed in more detail below. f_abs,route_ is a fraction between 0 and 1 that quantifies the amount of substance taken up via a specific route. PACEM considers oral, inhalation and dermal routes of exposure. Both EF_route_ and f_abs,route_ are substance specific and will vary from one application to the next. Values for these parameters need to be provided by the user. A more elaborate discussion on how to develop exposure fractions is given below. The summation in Eq. 3) is over the routes of exposure that are selected by a user.

### Combining product group survey data

To determine total aggregate exposure, the assessments for PCPs and HCPs are combined in the simulation. The HCP data set may be combined with either the “Dutch PCP” or the “European PCP” data sets. To do this, it is assumed that product use is completely uncorrelated between the different product groups (i.e., PCPs and HCPs). Joining data from the different surveys during simulation is done on the basis of sex and nationality only. First, a person is sampled from the model population based on the PCP usage data. This person has a particular PCP use profile. Next, a HCP use profile is added by randomly selecting an individual from the HCP data set of the same sex and nationality. The HCP use profile of this person is then merged with the PCP product usage data of the first sampled individual. It is thus assumed that there is a distinction in product usage between men and women and between nationalities, but not in other parameters, such as age.

Different PCP data sets (i.e., “EU PCP” and “Dutch PCP”) cannot be combined as they cover different products; thus, the PCP products available for simulation are filtered depending on the selected nationality (e.g., if “Netherlands” is selected, only the products in the Dutch PCP survey are available; if “France” is selected, only the products in the European PCP survey are available).

#### Exposure fractions

Exposure fractions summarize exposure that arises from product use to a number between 0 and 1 (0 representing no exposure, 1 representing exposure to all of the substance used during an event). Exposure fractions implicitly comprise information on the exposure duration, the release of the substance from the product, and contact between user and product. In PACEM, exposure fractions are user input which need to be derived outside PACEM. Exposure fractions may be derived by defining a standard exposure scenario for each product use. This scenario needs to be defined by making assumptions on exposure duration, personal behavior, substance and product properties. In such a scenario, dose D may be calculated for a specific amount (e.g., 1 g) of product. This may be done using custom models or dedicated exposure tools such as ConsExpo [28] or USEtox [29]. The exposure fraction is then obtained by dividing the dose D by the used amount of substance.

Note that the concept of an exposure fraction assumes that exposure is linear in the amount used. This assumption will not generally be exactly correct. E.g. in cases where saturation phenomena occur such as evaporation or dermal absorption, relative exposure will decrease with increasing amount used. Nevertheless, it is assumed to hold to a good approximation in general.

As an example, consider the inhalation exposure from a volatile cleaning agent in a liquid cleaner. As a scenario, the application of A = 1 g of cleaning agent in a small, low ventilation room (room volume V: 20 m^3^; ventilation rate q: 0.5 air changes/hour) is adopted. It is assumed that the substance evaporates immediately and disperses through the room. The user of the product remains in the room for 1 h and inhales the evaporated agent with an inhalation rate R_inhale_ of 1 m^3^/h. In this case, the amount D inhaled follows from:

4 $$D = \frac{{A \times (1 - e^{ - q \times t})}}{{q \times V}} \times R_{inhale} \approx 0.04g$$

The exposure fraction in this case is then taken to be 0.04/1 = 0.04.

Exposure fractions are used to determine systemic exposure. For dermal load, a similar concept is used. The so-called “retention factor” gives the fraction of amount of substance used that remains on the skin after use of a product. For retention factors PACEM provides default values for different products. For PCPs, the defaults are based on those suggested by the SCCS [1]. For HCPs retention factors as developed in [22] were included as default.

## PACEMweb tool

### PACEMweb interface

**Fig. 2:**
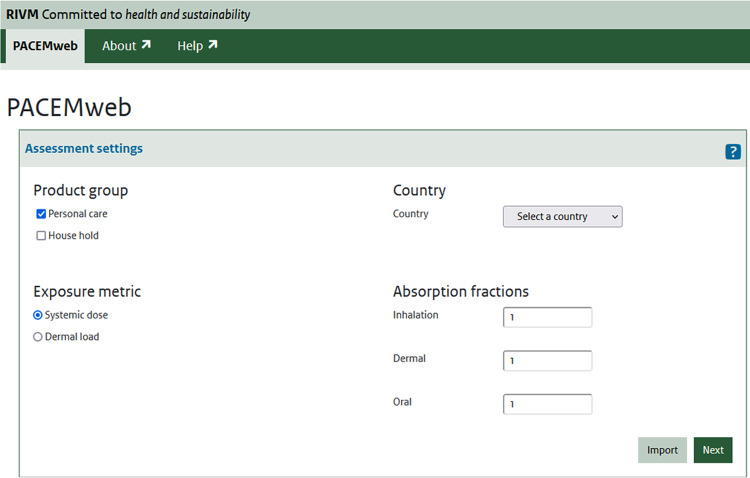
PACEMweb’s first input window: “assessment settings”. The assessment settings determine the product usage survey that is used in the simulation. Additionally, they determine whether exposure fractions or retention factors need to be specified in the next, “product settings” input window.
  When “systemic exposure” is selected as an output metric, absorption fractions need to be specified for each route of exposure included (i.e., inhalation, dermal, oral).
  In the second window (Fig. 3) the user selects which products should be considered and provides input on the concentration and exposure of those products. Concentration information is given as occurrence, i.e., as the % of products that actually contain the substance and the (distribution of the) concentration (mass/mass) of substance in the products that do contain the substance. Exposure fractions (when considering systemic exposure) or retention factors (when considering dermal load) are provided as mass fractions (g/g).

The web tool of PACEM is accessible via the url www.pacemweb.nl. Performing an exposure assessment with PACEM requires the user to set assessment settings and providing input values. Settings to be provided include (see Fig. 2):

- considered product group(s) (i.e., PCPs or HCPs or both),
- exposure metric (systemic dose or dermal load),
- the country for which the assessment is to be conducted. Options include the Netherlands, Germany, France, United Kingdom, Spain, or the latter 4 nations combined.

**Fig. 3:**
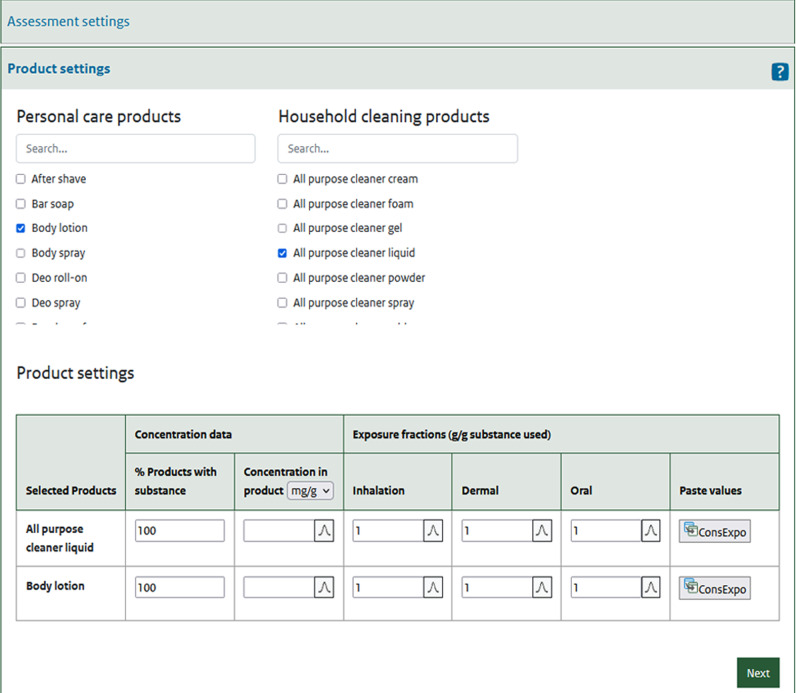
In the “product settings” window, the user selects products to include in the assessment and provides concentration and exposure information. Exposure fractions are entered manually or can be imported from the ConsExpo tool (www.consexpoweb.nl).
  After the specification of products and input values, the simulation is run on a web server. Feedback on the progress and completion are provided in a new window. After finalizing the simulation, users are presented with various options to report the simulation results. These options include:
- Time frame over which exposure is to be calculated. This may either be the average daily exposure over the 14 day simulation period, or the highest exposure on any day of the simulation period.
- Whether exposure for men, women, or both should be reported.
- Whether only the exposed individuals of the population should be reported, or both the exposed and non-exposed individuals.

**Fig. 4:**
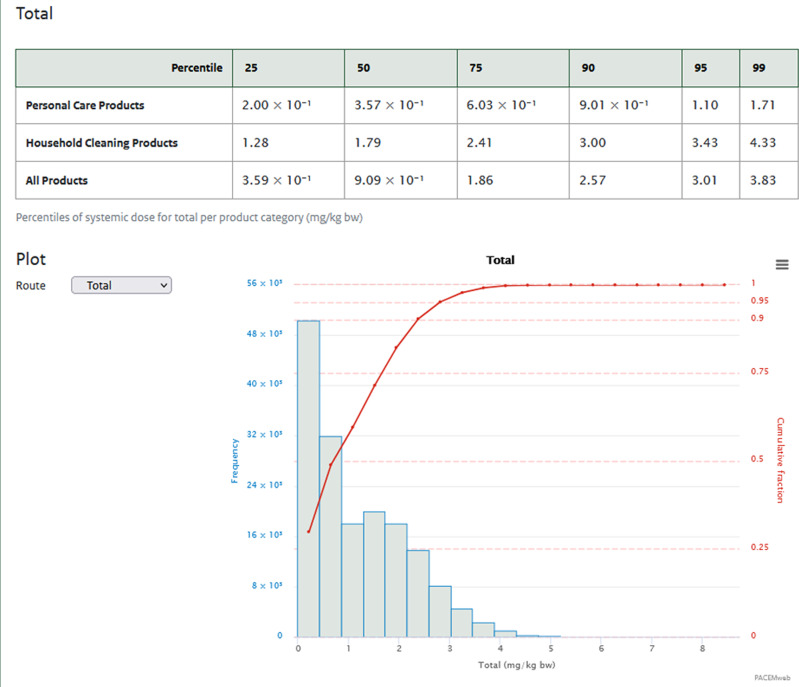
Analysis results. The results of the analysis are shown in summary. Depending on the selection made in the “Analysis settings” window, different measures of population exposure are presented as percentile tables as well as graphically.

Output is provided in a final form (Fig. 4). Exposure of the selected population is summarized as a table of percentile values, a histogram and cumulative plot of the (distribution of) exposure. This output includes different summary measures of population exposure that may be further used in chemical risk assessment.

## PACEM software implementation

PACEMweb is a ASP.Net Core 5.0 web application [30]. It has been built in MS Visual Studio [31], C#, using the following features: Razor; jQuery; SCCS; and Entity Framework code first. Hangfire [32] is used for performing background execution of simulations and clean-up tasks. Mathematical operations were implemented using the Math.Net Numerics library [33].

## Illustrative case study: exposure to methylisothiazolinone (MI)

To illustrate the application of the PACEM web tool, an exposure assessment for methylisothiazolinone (MI) was conducted. This assessment was an update of a previous risk assessment [21], updated with new data on MI concentrations in products [34]. MI is widely-used as a preservative. However, MI has been identified as a common cause of allergic contact dermatitis [21, 35–37]. To support the assessment of risk of sensitization, the aggregate dermal load (µg/cm^2^) was estimated using PACEM. MI concentration data were acquired for products of various product groups [34] (see Table S3). If more than one sample of a product in a product group contained MI, a log-normal distribution of the MI concentration was fit to the data and used as input in PACEM. If only a single measured product contained MI, a uniform distribution was assumed with upper and lower bounds that were ten times higher or lower than the measured MI concentration in that product. When deriving MI concentration distributions for HCPs, product groups representing products of the same type and exposure were combined, (i.e., liquids, sprays, and wipes) and taken to represent product groups of the same type and exposure. For instance, the concentration distribution for liquids in general, was used for all-purpose cleaner liquids, bathroom cleaner liquids, kitchen cleaner liquids, etc. Concentration information and input is presented in Table S3. Further details on this case study can be found in [34].

To illustrate the added benefit of the aggregate exposure estimates provided by PACEMweb, we also calculated the exposure to MI as the sum of the 95th and 99th percentiles of the exposures to single products in PACEMweb. The results of this simple additive (tier I) approach were compared to the aggregate exposure estimates.

## Aggregate exposure from MI: results

For the exposure assessment of MI, the short-time aggregate dermal load was chosen as the relevant output. In PACEM this is defined as the exposure on the highest day of each individual in the set of 14 days for which exposure is simulated. Results are shown in Table 1.

**Table 1:** Aggregated MI exposure expressed as maximum daily dermal load (μg/cm2) to PCP and HCP as calculated with PACEM for the Dutch population.

	Product group	Popula-tion	P25	P50	P75	P90	P95	P99
Tier 1	PCP	Both	—	—	—	—	7.5 × 10^−1^	9.6 × 10^−1^
	HCP	Both	—	—	—	—	2.6 × 10^2^	1.7 × 10^3^
	Total	Both	—	—	—	—	2.6 × 10^2^	1.7 × 10^3^
Aggregate	PCP	Men	0	0	3.4 × 10^−4^	2.6 × 10^-3^	4.5 × 10^−3^	1.1 × 10^−2^
	PCP	Women	0	2.8 × 10^−5^	9.5 × 10^−4^	5.7 × 10^−3^	1.5 × 10^−2^	0.14
	PCP	Both	0	1.2 × 10^−6^	6.8 × 10^−4^	3.9 × 10^−3^	9.0 × 10^−3^	9.5 × 10^−2^
	HCP	Men	0	0	1.2 × 10^−4^	1.9 × 10^−2^	6.6 × 10^−2^	0.62
	HCP	Women	0	0	0	1.8 × 10^−2^	6.8 × 10^−2^	0.83
	HCP	Both	0	0	0	1.8 × 10^−2^	6.7 × 10^−2^	0.74
	Total	Men	0	2.3 × 10^−4^	4.6 × 10^−3^	3.4 × 10^−2^	0.10	1.01
	Total	Women	7.2 × 10^−7^	3.5 × 10^−4^	6.0 × 10^−3^	4.4 × 10^−2^	0.12	0.88
	Total	Both	0	3.2 × 10^−4^	5.4 × 10^−3^	3.9 × 10^−2^	0.12	0.93

The exposure is shown as a percentiles table, giving exposure at pre-defined percentiles of the population. Exposure is reported for different sub-groups Men/Women, PCP/HCP/Total(HCP&PCP). The aggregate exposure was estimated using PACEM. Contrasted with this are results of a typical low tier (“Tier 1”) assessment, based on adding 95th exposure percentiles for all products. Adapted from [34].

Aggregate exposure to MI is higher via HCPs than via PCPs for the higher percentiles (i.e., P90 and higher) of the general population (i.e., both men and women). For percentiles lower than the 75th, exposure via HCPs is negligible and population exposure is dominated by PCPs. Overall, the highest dermal loads are, however, from HCPs. When comparing the dermal load between men and women, women seem to face higher exposures to PCPs than men, whereas exposures to HCPs are comparable. The total aggregate exposure is fairly similar between men and women for all reported percentiles.

The aggregate exposures estimated using PACEMweb was multiple orders of magnitude lower than the sum of the 95th and 99th percentiles of exposures to single products. The relevance of difference between the two modeling approaches becomes apparent when considering the acceptable exposure level (AEL) for MI. The 95th percentiles of the aggregate exposure estimates were generally lower than or similar to the AEL (7.4 × 10^−2^ μg/cm^2^) derived by Ezendam et al. [21]. In contrast, the straightforward tier I approach resulted in exposure estimates that substantially exceeded the AEL. These results thus emphasize that the probabilistic aggregate modeling approach provided by PACEMweb helps to refine simple additive tier I approaches.

## Discussion

The person-oriented approach to estimate aggregate consumer exposure adopted in PACEM leads to better informed and more realistic insight in human chemical exposure than crude, low-tier methods. Gosens et al. [18] as well as Dudzina et al. [20] demonstrated clearly that the low tier assessment methods lead to unrealistic high estimates of exposure that cannot straightforwardly be refined, lacking any insight in the level of uncertainty or conservatism of such estimates. In contrast, an assessment using PACEM allows for a much better gauging of the actual exposure in relation to an health-based limit value.

That being said, PACEM itself contains a number of significant uncertainties. First, there is the question of validity and representativeness of the product usage data. The surveys on the usage data were presented in peer-reviewed publications, providing confidence in the quality of the research that was conducted. However, it remains unclear whether the product usage data is representative for the population in any particular exposure assessment. For instance, the surveyed population for the Dutch PCP usage data set [11] is rather small (*n* = 516) which causes limitations on granularity (i.e., smallest size with which a population may be stratified).

Another concern is that product usage changes over time, which may impact the validity of exposure assessment in the future. Further developments on PACEM will include the expansion and updating of product usage data sets, to obtain larger sample sizes, more information on specific sub groups such as children and elderly, and changing trends, but also additional product groups such as e.g., painting products.

Quantitative validation or evaluation of an exposure model such as PACEM is not straightforward to conduct. Dudzina et al. [20] Delmaar et al. [19] and Karrer et al. [38] evaluated the results of a PACEM aggregate exposure assessment with biomonitoring data. Generally, PACEM predictions where within a factor 10 of the exposures estimated from biomonitoring data, and often better. Overall, results of PACEM were compatible with the biomonitoring data. However, such validation approaches are imperfect, since biomonitoring as a tool for exposure assessment comes with many limitations, such as the uncertainty in the relation between biomarker and external exposure, the bias in sampling strategy as time point of sampling and population sample size and representativeness. Nevertheless, these findings help build confidence in the plausibility of the approach.

The illustrative case study on MI exposure demonstrates how PACEM can be used to estimate aggregate exposure to a substance via the combined used of consumer products, and allows for a detailed analysis of the distribution of exposure. In particular, it provides information on the main contributions to aggregate exposure, and how the contribution may vary in a population. This can be particularly helpful when performing risk assessment of substances, but also when considering risk mitigation measures.

A practical limitation in the use of PACEM is the lack of built-in modeling capabilities to estimate exposure during product use events. Currently, exposure fractions need to be provided as user input. Exposure fractions simplify exposure to a linear relation between amount used and exposure. They need to be derived outside of PACEM. Recently, the ConsExpo tool has been updated with a feature to calculate and export exposure fractions for import in PACEM. This is a major improvement, but only addresses a part of the limitations. Future versions of PACEM are expected to feature a still tighter integration with ConsExpo, to provide a better workflow as well as a more refined estimation of exposure.

Another crucial aspect in the application of PACEM is the availability of detailed product concentration data. Generally, such information is sparse at best. For the US, the CPDat database [39] provides a useful source, but such a repository is not available for products on the EU market.

The surveys on consumer product usage underlying PACEM generally contain additional information on demographics of the surveyed population. Of this information, currently only information on sex, body weight and nationality is used. Including options for further stratification of the population into groups according to e.g., age or education level, could be a valuable addition to a future version of the tool.

Finally, it should be noted that PACEM only considers aggregate exposure from two groups of consumer products, HCPs and PCPs. Typically, other sources of exposure may be important as well. These may include other consumer products, exposure via the indoor or outdoor environment, occupational exposure or exposure via food stuff. While it seems difficult and impractical to follow a similar person-oriented approach as in PACEM, where population information on co-exposures at the individual level is used, methods to combine exposure assessments across these pathways and sources need to be developed. Methods to combine dietary and non-dietary exposure have been developed and applied [40, 41] but these are far from being generally applicable.

With regards to the consumer product groups, expansion of usage database in the coming years is expected. Studies on PCP usage among children, the use of painting products and an update of HCP use have either been planned or conducted already. Future versions of PACEM will contain this additional product usage information. Regular updates of PACEM, including expansion of the product usage database are foreseen.

## Conclusions

When considering the impact of a chemical’s exposure on human health, the total (or aggregate) exposure that stems from different sources should be considered, rather than only the exposures arising from each source separately. PACEM is a publicly available web tool that provides a method to realistically estimate aggregate exposure from the combined use of multiple consumer products. So far, such a tool has not been publicly available. Currently, PACEM enables exposure assessment for chemicals in HCPs as well as PCPs, but future versions may include additional product groups such as paints. This is a great improvement on the current state but methods to aggregate additional sources of exposure (such as occupational exposure, exposure via food and exposure from the environment) are also needed in the future.

### Supplementary information

Reporting Checklist

Supplementary tables

Supplementary information

## Data Availability

Data are available from the corresponding author on reasonable request.
