# Supplementary material for: PACEMweb: a tool for aggregate consumer exposure assessment
Source: J Expo Sci Environ Epidemiol. 2022 Dec 15;33(6):971–9. doi: 10.1038/s41370-022-00509-7 (PMC10733135; doi:10.1038/s41370-022-00509-7)
Supplement: Supplementary file 2 — Supplementary tables [file 41370_2022_509_MOESM2_ESM.docx]

Contents

[Survey information 2](#_Toc116560430)

[MI case input information 4](#_Toc116560431)

[Conversion European HCP data 5](#_Toc116560432)

List of Tables

Table S1 : Products included in the Dutch and EU surveys on PCPs.

Table S2: Summary of product usage surveys included in PACEM.

Table S3: Number of MI concentration measurements per product group (n) and input data used to calculate the aggregated exposure to MI in PACEM. The occurrence represents the percentage of measurements with a non-zero concentration. Adapted from [28].
