# Supplementary material for: PACEMweb: a tool for aggregate consumer exposure assessment
Source: J Expo Sci Environ Epidemiol. 2022 Dec 15;33(6):971–9. doi: 10.1038/s41370-022-00509-7 (PMC10733135; doi:10.1038/s41370-022-00509-7)
Supplement: Supplementary file 3 — Supplementary information [file 41370_2022_509_MOESM3_ESM.pdf]

Supplementary info

for

PACEMweb : a tool for aggregate consumer exposure assessment

Christiaan Delmaar, Roel Schreurs, Martine Bakker, Jordi Minnema, Bas Bokkers

Survey information

Table S1. Products included in the Dutch and EU surveys on PCPs.

	DUTCH PCP SURVEY	EUROPEAN PCP SURVEY
GENERAL HYGIENE	Deodorant	Deodorant Spray
	Perfume or Eau de toilette	Deodorant Roll On
	Shower gel	Eau de Toilette
	Bathing foam/oil	Eau de Parfum
	Toothpaste	Shower Gel
		Toothpaste
		Mouthwash
		Liquid Soap
		Bar Soap
SHAVING PRODUCTS	Shaving foam/gel/oil/soap	After Shave
	Aftershave	
HAIR CARE	Shampoo	Shampoo
	Conditioner	Rinse off Conditioner
	Hairspray	Hair Spray
	Other (gel, lotion, foam, wax)	Hair Styling Excl. Spray
	Hair dye	
SKIN CARE	Body lotion	Body Lotion
	Hand cream	Body Spray
	Day cream	Hand Cream
	Night cream	Face Moisturiser
	Facial cleaning lotion or tonic	
COSMETICS	Foundation	Liquid/Makeup Foundation
	Make-up remover	Lipstick
	Powder or rouge	
	Eye shadow	
	Mascara	
	Eye pencil	
	Eyebrow pencil	
	Lip pencil	
	Lipstick or lip gloss	
	Lip balm	
NAIL CARE	Nail polish	
	Nail polish remover	
TANNING PRODUCTS	Bronzers	
	Sunscreen	
	After sun	

Table S2. Summary of product usage surveys included in PACEM.

	**Dutch PCP survey**	**EU PCP survey**	**EU HCP survey**
N	516	23,232	1774*
COMPOSITION	men (59%), women (41%)	men (30%), women (70%)	men (39%), women (61%)
AGE RANGE	18-71	17-74	18+
COUNTRY	the Netherlands	France, Germany,UK, Spain	France, Germany, UK, Spain
YEAR	2012	2007-2008	2010-2011
NO. PRODUCTS	32	21	7 (various forms)**
CO-USE INFO	no	yes	no
FREQUENCY/DIARY	frequency	diary	frequency

* only of the four selected countries

** only the selected products

MI case input information

Table S3. Number of MI concentration measurements per product group (n) and input data used to calculate the aggregated exposure to MI in PACEM. The occurrence represents the percentage of measurements with a non-zero concentration. Adapted from [28].

MI concentration per product group						
	n	**Occurrence (%)**	**Geometric mean* (µg/g)**	**Geometric standard deviation* (µg/g)**	**Lower boundary* (µg/g)**	**Upper boundary* (µg/g)**
**PCP**						
Conditioner	13	15	1.6	6.0	n.a.	n.a.
Shower gel /foam/scrub	244	4	2.9	5.3	n.a.	n.a.
Eye pencil	1	100	n.a.	n.a.	0.05	5.0
Face cream day	30	3	n.a.	n.a.	9.0	900
Hand cream	49	27	1.5	3.8	n.a.	n.a.
Makeup remover	20	25	1.2	8.9	n.a.	n.a.
Mascara	3	33	n.a.	n.a.	0.32	32
Shampoo	226	8	4.0	5.6	n.a.	n.a.
Shaving gel	1	100	n.a.	n.a.	0.16	16
**HCP**						
Liquids	71	55	13	6.8	n.a.	n.a.
Sprays	46	48	9.5	11	n.a.	n.a.
Wipes	6	33	91	24	n.a.	n.a.

n.a.: Not applicable; either the lognormal (i.e. geometric mean and geometric standard deviation) or the uniform distribution (i.e. lower bound and upper bound) is chosen.

* rounded to two significant digits

Conversion European HCP data

Amounts

Measures from survey

spray	number of sprays	1-2 g per spray	taken from spray report (Delmaar and Bremmer, 2009). Pump spray 1-1.7 g/spray. Maximum value for a plant spray of 2.2 g/spray.
liquids	caps	40 g/cap	Measurement of 2 all purpose cleaners: 390 resp 400 mL/ 10 caps. Assume density of 1 g/mL
foams, crèmes, etc.	tablespoon	5 g	Wikipedia: 15 mL, assuming ~1g/mL
tablets	tablet	5-6 g/tablet	estimate
wipes and tissues	wipe	5 g/wipe	estimated from Wallmart product info (shipping weight) of 'Clorox disinfectant wipes' and 'DYMON Stainless Steal Cleaner Wipes'

Further assumptions
Uniform (U) distributions between the specified ranges
Amounts for 'more than X' have been assigned a range of [X – 2X]

Conversion of amounts

**measure**	**value**	**number**	**distribution**	**parameter 1 (g)**	**parameter 2 (g)**
spray	1	1 x	uniform	1	2
	2	2 x	"	2	4
	3	3 x	"	3	6
	4	4 x	"	4	8
	5	5 x	"	5	10
	6	> 6 x	"	6	12
cap	1	< 0.5	uniform	0	20
	2	0.5-1.0	"	20	40
	3	1.0-1.5	"	40	60
	4	1.5-2.0	"	60	80
	5	2.0-2.5	"	80	100
	6	2.5-3.0	"	100	120
	7	3.5-4	"	140	160
	8	4 - 4.5	"	160g	180
	9	4.5 - 5	"	180g	200
	7 (kitchen and bathroom cleaner)	3 caps or more	"	120	240
	10 (floor cleaner)	5 caps or more	"	200	400
tablespoon	1	<0.5	uniform	0	2.5
	2	0.5-1	"	2.5	5.0
	3	1-1.5	"	5	7.5
	4	1.5-2	"	7.5	10
	5	2-2.5	"	10	12.5

measure	value	number	distribution	parameter 1 (g)	parameter 2 (g)
	6	2.5-3	"	12.5	15
	7	>3	"	15	17.5
wipes	1	1	point	5	0
	2	2	"	10	0
	3	3	"	15	0
	4	4	"	20	0
	5	5	"	25	0
	6	>5	"	30	0
tablets	1	1	uniform	5	6
	2	2	"	10	12
	3	3	"	15	18
	4	4	"	20	24
	5	5	"	25	30
	6	>5	"	30	36

Frequency

The data is specified as a use frequency per product category (all-purpose cleaner, kitchen cleaner etc.) and the number of products used in the particular category (e.g. all-purpose spray, all-purpose liquid). The use frequency per specific product is not specified. For the conversion it is assumed that if multiple products are used in a specific category, the products are used with an equal frequency.
Thus, the use frequency for a product becomes:

$$\frac{use\ frequency\ category}{number\ of\ products\ in\ the\ category}$$

For the conversion of the use frequency from the questionnaire, the following conversion factors have been used:

Possible answers in survey	frequency	distribution	parameter 1	parameter 2
At least once a day	1 per day	point	1	0
Several times a week	2 - 6 per week	uniform	0.285	0.857
Once a week	1 per week	point	0.1428	0
Once every two weeks	1 per 2 weeks	point	0.0714	0
Once per month	1 per month	point	0.0333	0
Less than once a month	1 – 12 per year	uniform	0.00277	0.0333
Do not know	unknown	point	0	0
